# Nanomaterials meet surface-enhanced Raman scattering towards enhanced clinical diagnosis: a review

**DOI:** 10.1186/s12951-022-01711-3

**Published:** 2022-12-22

**Authors:** Kaisong Yuan, Beatriz Jurado-Sánchez, Alberto Escarpa

**Affiliations:** 1 Bio-Analytical Laboratory, Shantou University Medical College, No. 22, Xinling Road, Shantou, 515041 China; 2 Department of Analytical Chemistry, Physical Chemistry, and Chemical Engineering, University of Alcala, Alcala de Henares, 28802 Madrid, Spain; 3 Chemical Research Institute “Andrés M. del Río”, University of Alcala, Alcala de Henares, 28802 Madrid, Spain

**Keywords:** Nanotechnology, Cancer and pathogens, Medical diagnosis, SERS, Plasmonic metal, Nano-hybrid, Magnetic materials, SERS tags

## Abstract

Surface-enhanced Raman scattering (SERS) is a very promising tool for the direct detection of biomarkers for the diagnosis of i.e., cancer and pathogens. Yet, current SERS strategies are hampered by non-specific interactions with co-existing substances in the biological matrices and the difficulties of obtaining molecular fingerprint information from the complex vibrational spectrum. Raman signal enhancement is necessary, along with convenient surface modification and machine-based learning to address the former issues. This review aims to describe recent advances and prospects in SERS-based approaches for cancer and pathogens diagnosis. First, direct SERS strategies for key biomarker sensing, including the use of substrates such as plasmonic, semiconductor structures, and 3D order nanostructures for signal enhancement will be discussed. Secondly, we will illustrate recent advances for indirect diagnosis using active nanomaterials, Raman reporters, and specific capture elements as SERS tags. Thirdly, critical challenges for translating the potential of the SERS sensing techniques into clinical applications via machine learning and portable instrumentation will be described. The unique nature and integrated sensing capabilities of SERS provide great promise for early cancer diagnosis or fast pathogens detection, reducing sanitary costs but most importantly allowing disease prevention and decreasing mortality rates.

## Background

One of the biggest challenges of healthcare systems worldwide is the fast diagnosis of issues such as cancer and pathogens [1]. Early diagnosis can help decrease overall costs in treating such issues and most importantly, decrease the mortality rates. Yet, target biomarkers are present at extremely low levels in early cancer stages [2]. Pathogens can be grown *in-vitro* to obtain sufficient cells, increasing the detection sensitivity, yet such procedures are time-consuming. The balancing between fast discrimination and sensitive detection of pathogens is the key to providing effective guidance in antibiotic therapy [3, 4]. In addition, disease diagnosis is also hampered by the inherent complexity of biological media, which prevents direct detection without sample processing [5, 6].

**Fig. 1:**
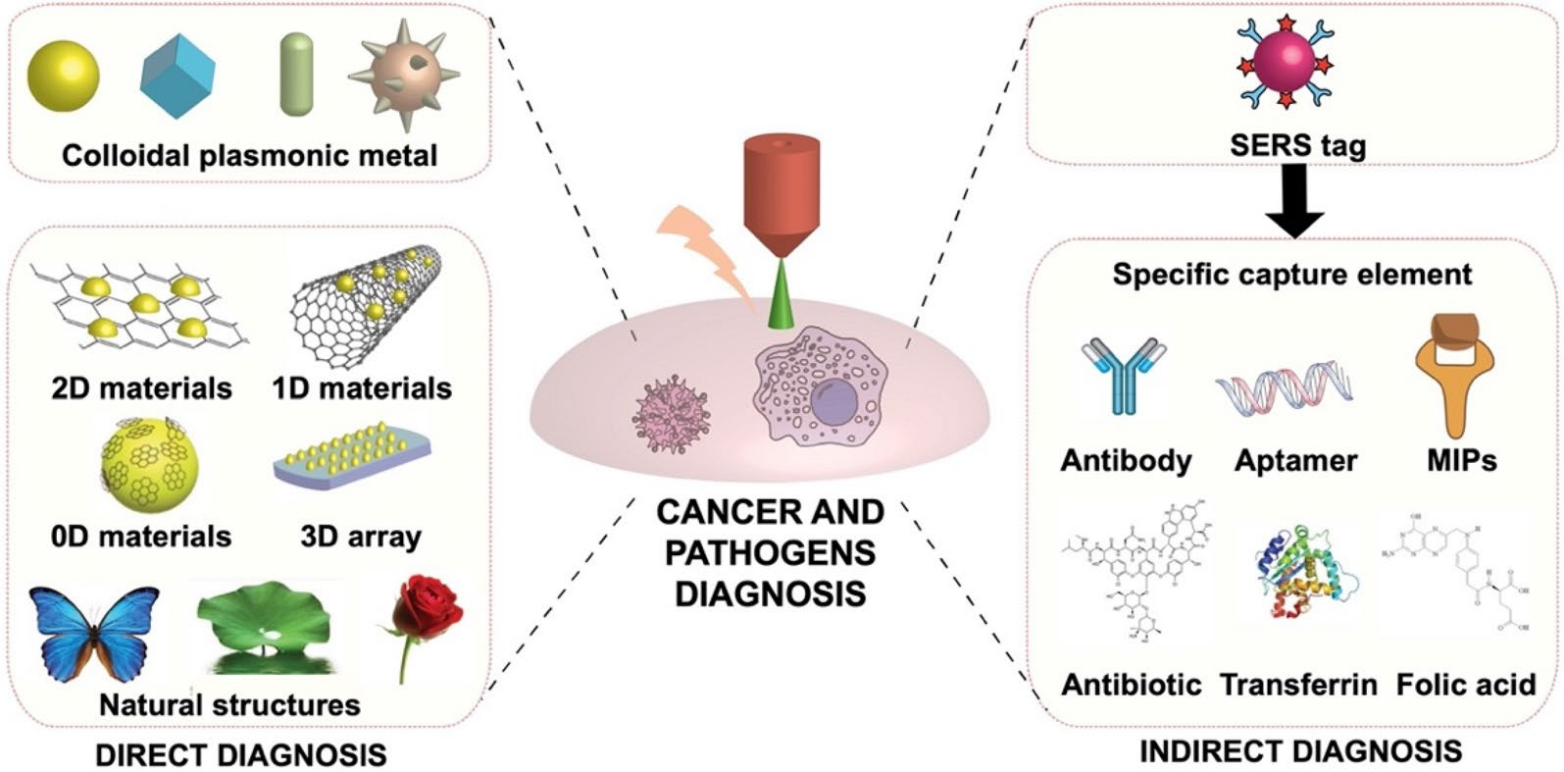
Schematic illustration of SERS strategies for cancer and pathogens diagnosis

SERS is a very promising tool for the direct detection of biomarkers in disease diagnosis applications. The technique relies on the enhancement of inelastic light scattering molecules (or analytes) attached or combined with plasmonic metals or semiconductor materials [7–9]. Since the first discovery of the SERS in 1974, researchers have proven its suitability in analytical science, ranging from environmental monitoring to biological/biomedical detection [10–14]. The broad scope of operations and applications, along with the high sensitivity, rich molecular “*finger-print*” information (specificity), in-situ detection, and non-destructive nature [15–17], have paved new ways for SERS application in cancer and pathogens diagnosis [18, 19]. Indeed, cancer and pathogenic cells and related biomarkers are mostly composed of proteins or other organic molecules with inherent Raman signals, allowing for direct detection and target discrimination based on the different Raman responses (*fingerprints*) of their compositions. Other key features of SERS include high sensitivity and fast detection, allowing disease diagnosis even at a signal cell/molecular level [20–22]. The type, size, and shape of the SERS substrate used for enhancing the Raman signal will exert a strong influence on a given biological application. This is of extreme importance to also address the critical challenge of non-specific interactions with co-existing substances in the biological matrices, which can hamper sensitivity. SERS substrates can be classified into two main groups according to the enhancement mechanism: (a) electromagnetic enhancement (EM) or (b) chemical enhancement (CM). EM derives its enhancing ability from electromagnetic effects on nano-structured metallic surfaces, reaching an enhancement factor (EF) of 10^4^–10^10^. CM is caused by charge transfer, with a lower degree of the overall Raman enhancement (2 orders) [23, 24]. The integration of both mechanisms into hybrid SERS substrates shows considerable promise for synergetic enhancement and metal protection, achieving fast and reliable disease diagnosis in biological samples, and avoiding interferences in most cases [25, 26]. Using ingenious designs such as magnetic separation or introducing SERS tags labeled with recognition elements, the disease diagnosis can be conducted with high selectivity [27, 28]. Figure 1 illustrates a schematic summary of the previously described strategies.

As can be seen in Fig. 1, the main advantage of SERS in cancer and infectious diseases diagnosis is the ability for the direct and fast detection of the cells or related biomarkers by exploiting the specific Raman fingerprint in discrimination, which origins from the different Raman-active molecules of the cell lines [29–32]. Indeed, disease diagnosis conducted in this way can provide a rich spectrum containing information about the analyte, allowing for further in-depth studying of related diseases at the molecular level [33–35]. The major challenge of current direct detection is the low content and small Raman scattering cross-section of Raman active chemicals on the cell surface, leading to weak SERS signals and limited sensitivity in disease diagnosis [36, 37]. Therefore, different substrates for signal enhancement have been explored [38]. Colloid plasmonic metals with EM effect such as gold and silver nanoparticles have been the traditional choices [39, 40]. To increase the efficiency of the Raman enhancement, nanomaterials of different shapes such as nanostar, nanorod, nanocube, etc., with abundant branches or edges, were explored [41–43]. In addition, semiconductors with CM effect, such as graphene, MoS_2_, etc., have been also introduced to further improve SERS properties [44–46], as well as for protecting active tags from degradation, or to impart further surface functionalities [47, 48]. Apart from sensitivity, signal stability is another challenge in the direct diagnosis of cancer and pathogens. Colloidal SERS substrates prepared by simply mixing the tag with the biological sample are prone to the generation of heterogeneous and random *hot-spots*, which leads to differences in enhancement factors to the Raman active molecules and unstable signals [12, 49, 50]. 3D-ordered SERS substrates are a convenient alternative to avoid such problems [51–54]. Natural creatures possess ordered nanostructures, endowing them with specific functionalities such as hydrophobicity, strong adhesive force, etc. [55–57]. Such natural nanostructures also are widely used in fabricating low-cost 3D solid substrates for SERS [58, 59]. In addition, cancer and pathogens diagnosis using SERS is hampered by interferences from the complex biological matrix used, which is also a critical point in clinical application. A typical solution relies on the introduction of an indirect SERS strategy, involving a Raman reporter, SERS active nanomaterial, and specific recognition element to fabricate a “three-in-one” SERS tag, thus providing a strong SERS signal, and realizing specific affinity with target cells even in a complex matrix [60–62].

In this review, we will describe recent advances and prospects in SERS-based approaches for cancer and pathogens diagnosis. As a distinct point, this review will cover both cancer and pathogens diagnosis, and most importantly, will focus on different strategies to improve SERS performance in a view to providing the readers with important tools to improve overall credibility and analytical performance with diagnosis purposes. We will discuss first direct SERS strategies for sensing cancer and pathogens cells and related biomarkers, including the design of different SERS substrates such as plasmonic, semiconductor structures, and 3D order nanostructures for signal enhancement. Secondly, we will illustrate recent advances for the indirect diagnosis of cancer and pathogens by the introduction of SERS tags, which combine SERS active nanomaterials, Raman reporters, and specific capture elements into *“three-in-one”* probe units. Thirdly we will be, for the first time, a critical overview of challenges and solutions to translate the existing proof-of-concept applications into real clinical settings, covering from portable SERS detection to machine learning to discriminate the rich but complex SERS spectra. Further challenges will be briefly discussed in the conclusions. The unique nature and integrated sensing capabilities of SERS provide great promise for early cancer diagnosis or fast pathogen detection, reducing sanitary costs but most importantly allowing disease prevention and decreasing mortality rates.

## Direct SERS detection of cancer and pathogens

Direct SERS sensing of target analytes is achieved by direct attachment to the SERS substrate, obtaining both qualitative (“*finger-print*” of spectra) and quantitative (signal intensity) determination [63]. A major superiority of direct SERS over other strategies is the obtention of a rich spectrum with “*finger-printing*” information of the target molecules without the need for further labeling [64–66]. In the diagnosis of cancer and pathogens, this strategy can provide in-depth information from biomarkers, cells, and/or their interactions, thus providing the possibility for revealing target compositions and disease mechanisms [67–70]. Yet, the low content of Raman active molecules on the cell surfaces, the low concentrations of related biomarkers, and the complexity of biological fluids, results in low signal intensities, hampering this detection. In addition, the heterogeneous aggregation of the SERS active nanoparticles can lead to low reproducibility. The key to achieving high sensitivity and reproducibility is to select and tailor the composition of the SERS substrates.

### Plasmonic metal nanoparticles as SERS substrates

**Fig. 2:**
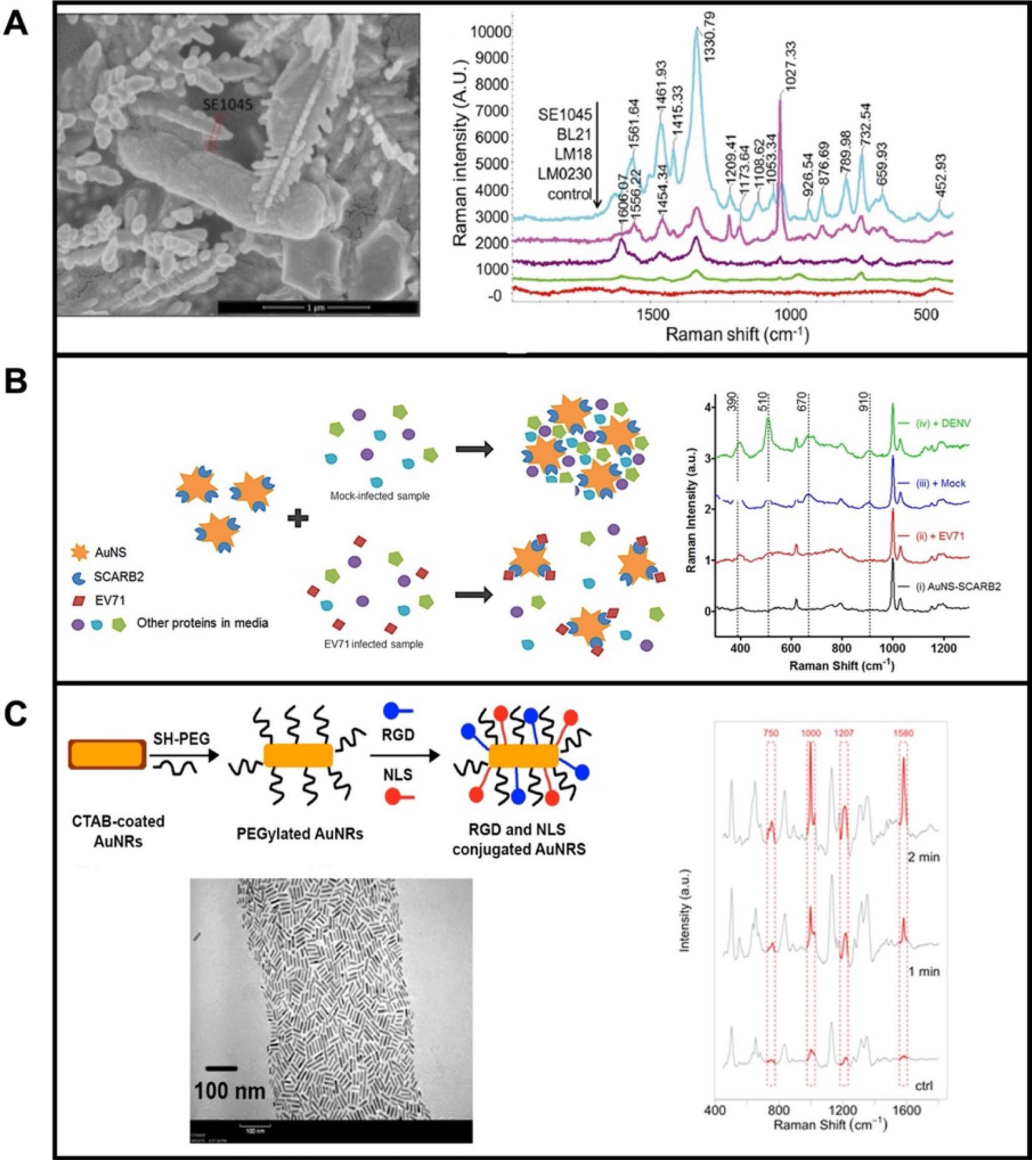
**A** Direct SERS sensing of bacteria using Ag dendrites as Raman substrates: SEM images of the *Salmonella enterica* (SE1045) mixed with Ag dendrites (left) and SERS spectra of different bacteria strains using Ag dendrites as substrate. **B** Gold nanostars as SERS substrate for direct diagnosis of enterovirus (EV) 71: schematic of EV71 SERS sensing based on anti-aggregation of gold nanostars and Raman spectra of SCARB2 modified Au nanostars added to mock-infected cell culture in the presence and absence of EV71 and the presence of DENV. **C** Gold nanorod as SERS substrates for studying cancer cell death mechanisms: schematic of the sensing strategy, TEM images of the modified nanorods, and SERS spectra collected of a single HSC-3 cell under NIR laser exposure for 1 and 2 min. Reprinted with permission from ref. [79] (**A**); [80] (**B**) and [87] (**C**)

Direct SERS sensing with i.e., silver, gold, and copper nano colloids involves the direct mixing with the sample to induce the generation of biomarker-nanoparticle or cell-nanoparticles aggregates. The as-generated SERS spots will increase the intensity of the Raman signal. Different metal nanomaterials with variable shapes have been explored, including nanoparticles [71, 72], nanorods (NRs) [73, 74], nanostars [75, 76], nanocubes [77, 78], etc. For example, Wang et al. [79] synthesized silver dendrites for direct SERS detection and discrimination of *Salmonella enterica* in the presence of *Escherichia coli* (see Fig. 2A). Thus, the scanning electron microscopy (SEM) images show the morphology of the silver dendrites-*Salmonella enterica* complex (left part). Further mapping of the specific peaks allows for the specific detection (Raman shift, 1332 cm^−1^) of *Salmonella enterica* with a limit of detection (LOD) of 10^4^ CFU mL^−1^ (see middle part). In addition, such SERS substrates have higher enhancement activity for Gram-negative bacteria than for Gram-positive bacteria, avoiding thus interferences for selective discrimination (right part).

Reyes et al. [80], developed a SERS strategy for direct and rapid determination of Enterovirus 71 (EV71) using Au nanostars colloids as plasmonic substrates (Fig. 2B). The surfaces of Au nanostars were modified with EV71 affinity protein, and recombinant scavenger receptor class B member 2 (SCARB2) protein, for the further specific detection of the target. Nonspecific proteins from the sample would induce the aggregation of SCARB2-modified Au nanostars, producing Raman signals (peaks at 390, 510, 670, and 910 cm^−1^). While EV71 viruses are presented in the biological sample, SCARB2- modified Au nanostars can be combined with EV71, followed by the anti-aggregating of Au nanostar colloids and the diminishing of the Raman peaks (left part). Figure 2B revealed that peaks at 510, 670, and 910 cm^−1^ only disappear in the presence of EV71 virus while not in the presence of Dengue virus (DENV) or mock-infected cell culture supernatants, which proved the high specificity of the proposed method.

Plasmonic nano colloids such as Au nanorods (AuNRs) [81, 82], multilayered Au nanoshells [83], Au nanostars [84], etc., not only show excellent SERS activity but also highly efficient photothermal conversion upon near-infrared (NIR) irradiation, thus have been used in cancer-related photothermal therapy. The *in-situ* detection and time-dependent changes of Raman *fingerprints* of target molecules in such substrates are also advantageous to explore the mechanism of cancer-related biological and chemical processes [85, 86]. Ali et al. [87] employed AuNRs with high photothermal conversion efficiency to study its underlying photothermal effect (Fig. 2C). Using a seedless method, AuNRs with average sizes of 25 nm × 6 nm were obtained. Further surface modification of PEG, Arg-Gly-Asp (RGD), and nuclear localization signal (NLS) improve the biocompatibility, cell uptake, and targeting ability of the AuNRs (Fig. 2C). After cell (HSC-3 cell) uptake, a NIR laser was used to irradiate the cells at different time intervals, leading thus to an increase in the temperature. Simultaneously, the Raman spectra were recorded to monitor molecular changes from the AuNRs containing microenvironment. As depicted in Fig. 2C, peak intensities at 750, 1000, 1207, and 1580 cm^−1^ increased after exposure to the NIR laser. From the changes in SERS peaks, the authors deduced related conclusions including “phenylalanine increases in the microenvironment (perturbation of phenylalanine metabolism) during photothermal therapy,” and “apoptotic cells” (*cytochrome c*-mediated apoptosis) increase during thermal heating.” Combined with metabolomics and proteomics experiments, this work demonstrates the potential of AuNRs for photothermal therapy at and from the molecular level. Other plasmonic colloid metals used as SERS substrates for direct sensing of cancer, pathogens, and related biomarkers are summarized in Table 1.

**Table 1:** Plasmonic colloid metal as SERS substrate for direct sensing of cancer, pathogens, and related biomarkers

Target analyte/cell	Interaction mode	Peaks (cm^−1^)	Sensitivity	Refs.
Ag NPs				
*Staphylococcus aureus*	Aptamer as capture elements	735, 1337, 1458	1.5 CFU mL^−1^	[88]
Prostatic cancer (DNA/RNA) biomarkers	Electrostatic interaction	560, 742, 788, 913, 1035, 1180, 1247, 1334, 1457, 1539, 1632	100 copies of input RNA	[89]
*Mycobacterium bovis BCG*, *Mycobacterium tuberculosis Staphylococcus aureus* *Staphylococcus epidermidis* *Bacillus cereus*	In-situ coating	731, 1031, 1326, 1463	10^2^ CFU mL^−1^	[90]
Prostate cancer (RNA) biomarkers	Electrostatic interaction	742	100 synthetic RNA copies	[91]
AuNPs				
Colorectal cancer biomarkers	–	724, 1263, 1574	Above 90%	[92]
Au nanostars				
Protein Kinase A activity for cancer screening	–	725, 1395	–	[93]
Au/Ag bimetallic NPs				
*Escherichia Coli*, Salmonella typhimurium, *Bacillus subtilis*	–	656, 730, 958, 1082, 1324, 1581	–	[94]
Core–shell Au@Ag NPs				
*Escherichia coli* *Staphylococcus aureus*	Interaction between negative (bacteria) and positive (PEI)	655, 729, 958, 1328, 1583 (*E. coli*) 733	10^3^ CFU mL^−1^	[95]
Exosomes as cancer biomarkers	Electrostatic attachment and in situ formation	668, 707, 786, 1179, 1490, 1563 (B16F10) 645, 1000, 1211, 1326, 1381, 1563, 1592 (RBC) (RBD)	> 90%	[96]

### Nanomaterials incorporated plasmonic metal nano-hybrids as SERS substrates *2D nanomaterials*

**Fig. 3:**
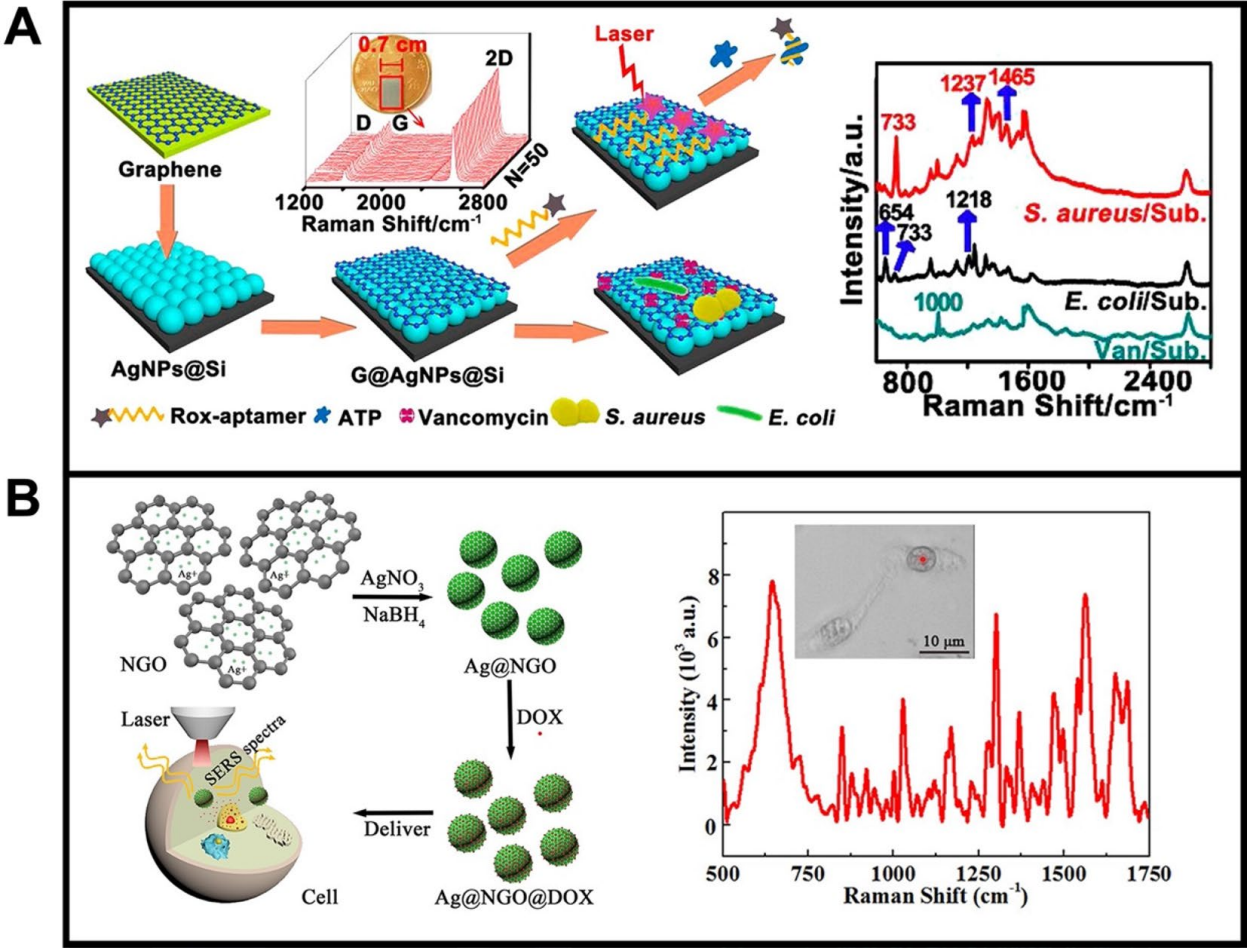
**A** Graphene-silver nanoparticles-silicon (G@AgNPs@Si) as SERS substrate for bacteria discrimination: Schematic illustration of the strategy and corresponding SERS spectra for *Staphylococcus Aureus* and *Escherichia coli*. **B** Nanosized graphene oxide coated with silver nanoparticles (Ag@NGO) as SERS substrate for intracellular detection: schematic illustration of the synthesis and corresponding intracellular SERS biosensing and SERS spectrum of HepG-2 after incubation with Ag@NGO nano-hybrid (right). Reprinted with permission from ref. [101] (**A**) and [99] (**B**)

The convenient marriage of plasmonic metal and 2D nanosheets led to novel platforms with high SERS activity, high stability, low background signal, and multi-functionality with a broad scope of applications [97–100]. Meng et al. [101] developed a graphene-silver nanoparticles-silicon (G@AgNPs@Si) sandwich SERS chip, as depicted in Fig. 3A. AgNPs were grown in situ on silicon substrates (Si), followed by wrapping Ag surfaces with graphene (G) monolayer. Such G@AgNPs@Si nano-hybrids show synergistic effects including electromagnetic enhancement (Si-reflected plasmon resonance; AgNPs-scattered plasmon resonance) and chemical enhancement (graphene-based charge-transfer resonance), which results in superior SERS activity. The chip was modified with vancomycin for direct capture and sensing of *Staphylococcus Aureus* and *Escherichia Coli*. Figure 3A shows the representative Raman peaks of *Staphylococcus Aureus* (1237 cm^−1^ and 1465 cm^−1^) and *Escherichia Coli* (654 cm^−1^ and 1218 cm^−1^) bacteria, illustrating the selectivity of the protocol. Zeng et al. [99] fabricated a SERS substrate using nanosized graphene oxide coated with silver nanoparticles (Ag@NGO). The NGO showed superior chemical inertness and optical penetration (Fig. 3B), along with a uniform size (~ 20 nm diameter) and round morphology, to facilitate intracellular uptake in further biomedical sensing applications (middle). Specific peaks such as 1651, 1564, 1302, 1030, 850, and 642 cm^−1^ are obtained in the SERS spectra of HepG-2 cancer cells (right). Such Raman signals are only produced inside the cells, indicating the successful cell penetration of the Ag@NGO nanoparticles.

An updated list of recent 2D nanomaterials such as BP, g-C_3_N_4_, MoS_2_, h-BN, and WS_2_ used in connection with plasmonic metals as SERS substrates for cancer and pathogens diagnosis are listed in Table 2.

**Table 2:** 2D materials plasmonic metal as SERS substrate

2D nanomaterial	Nano-hybrid	Hybrid structure	Target analyte/cell	Refs.
Black phosphorus (BP)	BP-Au filter		*Staphylococcus aureus* *Escherichia coli* Listeria	[102]
	BP-Au nanosheets		HepG2 cells	[103]
			Breast tumors	[104]
			Fibroblasts	[105]
g-C_3_N_4_	g-C_3_N_4_ nanosheet Au@Ag		HeLa cell	[106]
	Mesoporous g-C_3_N_4_		6-thioguanine	[107]
MoS_2_	MoS_2_-Au nanosheets		Fibroblasts	[105]
	AuNPs/GO@MoS_2_/AuNPs		DNA	[108]
Boron nitride (BN)	Cu@HG@BN nanosheets		In vitro microRNA sensing	[109]
	AuNS@hBN		Quorum sensing of bacterial biofilms	[69]
WS_2_	WS_2_-Au nanosheets		*Salmonella DT104 Salmonella Typhi*	[110]

#### 1D nanomaterials

As an important member of the 1D materials family, carbon nanotubes (CNTs) have broad applications ranging from extraction and enrichment to biological sensing due to their unique structure and properties [111–113]. CNTs compromise single-wall carbon nanotubes (SWCNTs) and multi-wall carbon nanotubes (MWCNTs), which are composed of sp^2^-hybridized carbon atoms, exhibiting high chemical stability [114], large surface area [115] and biocompatibility [116]. The inherent tubular morphology of CNTs allows for the direct growth of plasmonic metal on their surfaces without any pretreatment. In addition, the as-deposited nanoparticles are restricted to the nanoscale size due to the small diameter of CNTs [117]. Researchers have also exploited the inherent stability of SWCNTs against photo-bleaching to design a myriad of radiometric SERS nanosensors [118, 119]. The use of CNTs-based hybrid as SERS substrates has been successfully illustrated for the direct detection of explosives and other toxic molecules [120, 121], yet applications for detection in the biomedical field remain unexplored.

#### 0D nanomaterials

**Fig. 4:**
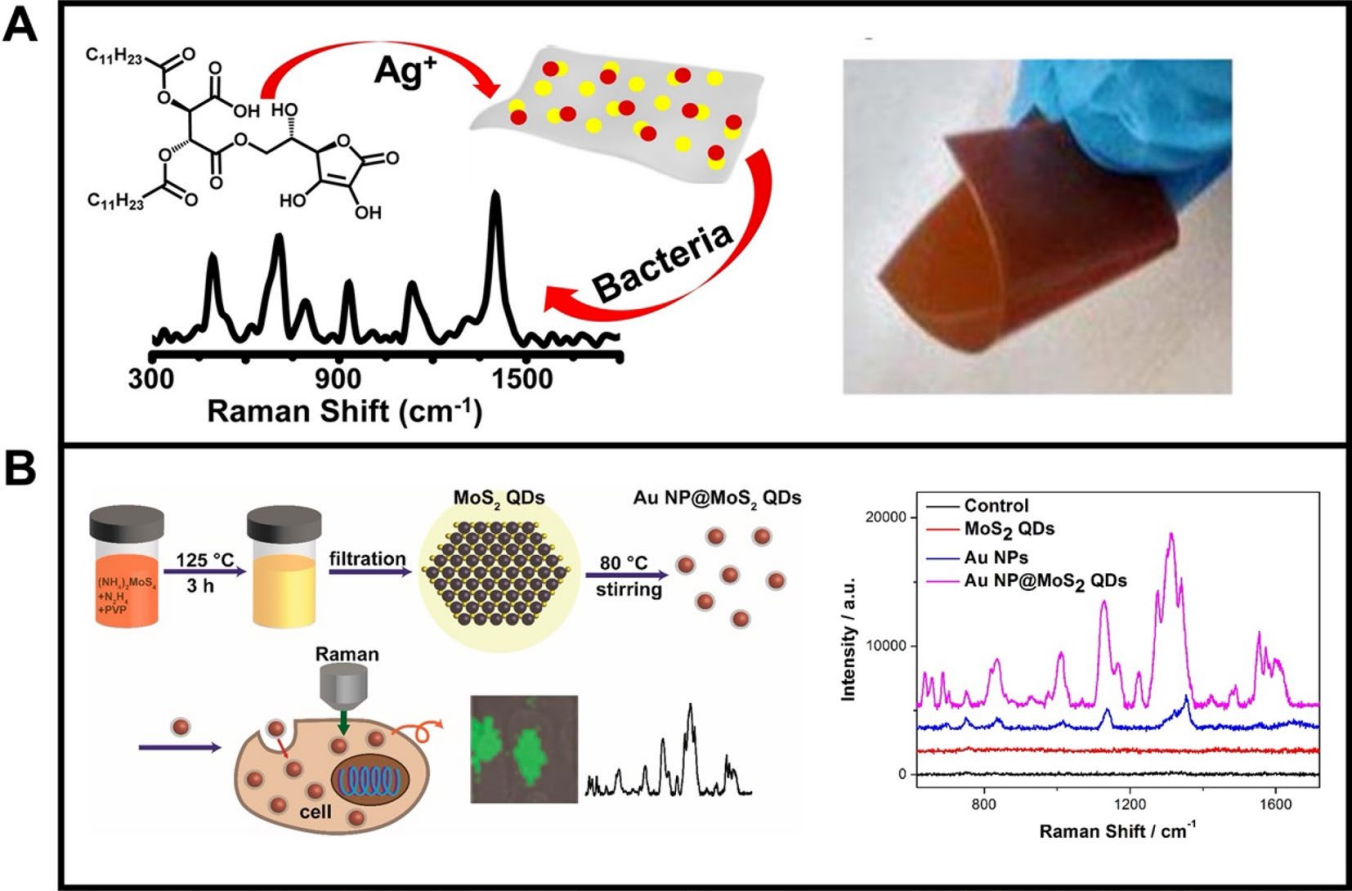
**A** Carbon-Dot/Silver nano-hybrid as SERS films for bacteria detection: schematic illustration of the synthesis of C-dot-Ag-NP-PDMS films, photography showing the flexibility of the film and SERS spectrum of 10^4^ CFU/mL of *Pseudomonas Aeruginosa* **B** AuNP@MoS_2_ quantum dots nano-hybrid for SERS sensing of cancer cells: schematic illustration of the synthesis of AuNP@MoS_2_ quantum dots nano-hybrid and related biological SERS application and SERS spectra of 4T1 cells mixed with MoS_2_ QDs (red line), AuNPs (blue line) and AuNP@MoS_2_ nanohybrid (violet line). Reprinted with permission from ref. [124] (**A**) and [125] (**B**)

Compared to traditional 2D nanosheets, 0D materials show unique advantages, such as higher adsorption abilities due to the larger specific surface areas [122] or higher SERS activity derived from Van Hove singularities in the density of states [123]. Bhunia et al. [124] reported the development of carbon-dot/silver-nanoparticle (C-dot-Ag-NP) PDMS SERS films for bacteria sensing. For preparation, the PDMS precursors and ascorbic acid are heated at 60℃ to form the PDMS film. The encapsulated ascorbic acid function as a carbon precursor for C-dot formation by reduction with silver acetate at 125 °C (Fig. 4A). Such design results in the generation of uniform C-dots with diameters between 2 and 5 nm, along with flexible nature. The integration of AgNPs and C-dots played a critical role in the high SERS activity, whereas no Raman peaks were obtained using PDMS films containing only AgNPs or C-dots. The C-dot-Ag-NP-PDMS films were applied for the detection of *Pseudomonas Aeruginosa*, as well as distinguishing between *Bacillus Aureus*, and *Erwinia Amylovora 238* bacteria. Fei et al. [125] employed Au NPs@MoS_2_ quantum dots (Au NP@MoS_2_ QDs) nano-hybrids as SERS substrates for cancer cell imaging (Fig. 4). As indicated in the TEM observation, such nano-hybrid possess core–shell structures with an ultrathin MoS_2_ QDs-coating. Such AuNPs@MoS_2_ QDs nano-hybrids were further used for 4T1 cell imaging, which showed much higher SERS intensity compared with single MoS_2_ QDs or AuNPs.

### 3D-ordered solid nanostructures as SERS substrates

One obstacle that traditional plasmonic nanoparticle-based SERS sensing is the need to overcome the low repeatability of the signal intensity. Thus, the aggregation of the nanoparticle colloids is random and heterogeneous, resulting in different SERS enhancement abilities to the attached target analytes even in the same sample [126, 127]. 3D solid nanostructures hold great promise to solve this drawback. Indeed, the composition of 3D nanostructures can be tailored to obtain periodic nanostructures with specific nanogaps at fixed positions, resulting in good repeatability and sensitivity to SERS sensing [12, 128, 129]. The synthesis is conducted by bottom-up self-assembly methods [130, 131] or top-down nanolithography techniques [132, 133]. Nanosphere lithography (NSL) is a widely used approach to synthesized 3D surfaces. NSL consisted of the formation of a monolayer of nanospheres with uniform sizes on a flat surface, followed by the deposition of a noble metal film using thermal evaporation or electron beam deposition. Next, a sonication or stripping process is used to remove the resulting hybrid nanospheres [134–136]. Electron beam lithography (EBL) is widely used for top-down fabrication of periodic nanostructures with arbitrary shapes and tunable interparticle nano-gaps in a small distance, which is key for both repeatability and high active SERS enhancement [137, 138]. 3D nanostructures with specific designs have been used as SERS substrates for cancer and pathogens detection, including Ag arrays, Ag nanoring cavities, Au octupolar meta structures, etc. Typical examples are listed in Table 3.

**Table 3:** 3D ordered nanomaterials as SERS substrates for direct sensing of cancer, pathogens, and related biomarkers

Nanomaterial	Schematic of the SERS substrate	Target analyte/cell	Interaction mode	Refs.
AgNPs arrays		*Lactobacillus plantarum*, *Escherichia coli*	Vancomycin as capture element	[139]
Ag nanoring cavities		DNA base (adenine)	Direct contact	[140]
Au octupolar metastructures		*Brucella*	Phage as capture elements	[141]
Silver nanorod (AgNR) array on PDMS substrate		*Pseudomonas aeruginosa*	Direct contact	[142]
AgNPs on a mesoporous silicon substrate		*Escherichia Coli Staphylococcus Epidermidis*	Direct contact	[143]
Ag film substrate		Circulating tumor cells (CTCs)	Direct contact	[144]
Highly branched AuNPs on a silicon wafer		Carcinoma cancer cells	Direct contact	[35]
Ag nanorod array		Respiratory viruses (RSV) strains A2, A/Long, and B1	Direct contact	[145]
Ag-Cr coated nanovoid structure		Cytochrome C	Direct contact	[146]
Au grating		DNA (discriminated DNA-DNA interaction)	Complementary between single oligonucleotide	[147]
Ag-coated nanowire arrays		*Bacillus anthracis* spores	Direct contact	[148]
Multilayered metal–insulator- metal nanostructures		Breast cancer	Grown on the substrate directly	[149]
Au ordered superlattices		Kynurenine, tryptophan, and purine derivatives	Direct contact	[150]

**Fig. 5:**
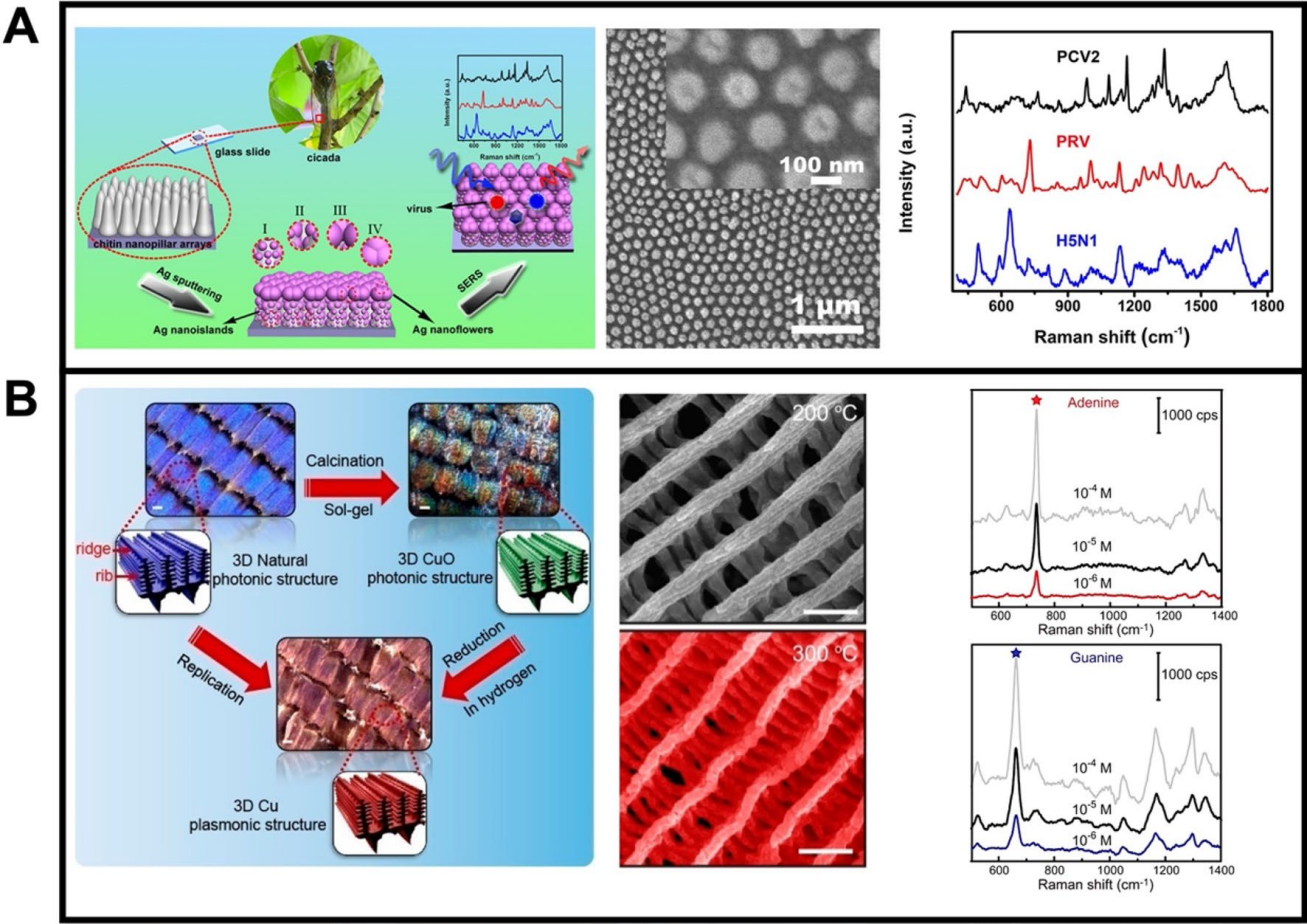
**A** Bio scaffold arrays of cicada wings as 3D templates for the fabrication of biomimetic SERS substrate: schematic of the fabrication process and virus detection, SEM images of the resulting 3D biomimetic Ag-decorated substrates and SERS spectra of PCV2, PRV, and H5N1 illustrating the Raman enhancement. **B** Schematic illustration in the fabrication of 3D Cu plasmonic structures photonic structures using butterfly wings, SEM characterization of the substrate, and SERS spectra of DNA base molecules, (adenine and guanine), acquired using the Cu decorated butterfly wings substrates. Reprinted with permission from ref. [157] (**A**) and [158] (**B**)

Natural structures have been explored for the preparation of 3D surfaces, further evaluating the potential for SERS sensing. For instance, hierarchical nanostructures impart lotus leaf and rose petals with surface superhydrophobicity [151, 152], photonic crystals are present in butterfly wings [153], and the high density of nano-size tentacles in toepads make gecko exhibit strong adhesive force to the wall [154], etc. Such natural periodic 3D structures can function as excellent bio-templates for decoration with noble metal NPs to fabricate SERS substrates [59, 155, 156]. Shao et al. [157] used cicada wings as bio scaffold arrays for decoration with AgNPs, forming thus 3D SERS substrates with hierarchical nanogaps. As depicted in Fig. 5A, the decoration of the bio-template is conducted by ion-sputtering techniques. SEM observation illustrates the periodic 3D nanostructure, resulting in a high reproducibility in target sensing, along with nanogaps to generate more hot spots for sensitive detection. Lateral real application has used this bio-SERS substrate for the discrimination among PCV2, PRV, and H5N1 viruses due to the specific Raman spectrum and discrimination analysis. Tan et al. [158] exploited the photonic crystals of butterfly wings as bio-templates for modification with Cu superstructures (Fig. 5B). However, the original chitin and protein from butterfly wings caused SERS signal impurities and fluorescence interferences in target sense. To solve such an issue, the authors employed a reduction method using H_2_ to obtain Cu decorated wing SERS substrate with low background signals. The SEM images of Fig. 5B show the morphology of the biotemplate after the reduction process, with no obvious changes in the morphology after reduction by H_2_ in elevated temperature. Compared with unmodified butterfly wings, Cu butterfly wings showed clean SERS spectra after the reduction process, with adequate adenine and guanine (DNA) detection, with excellent sensitivity at a low cost.

### Magnetic SERS substrates

**Fig. 6:**
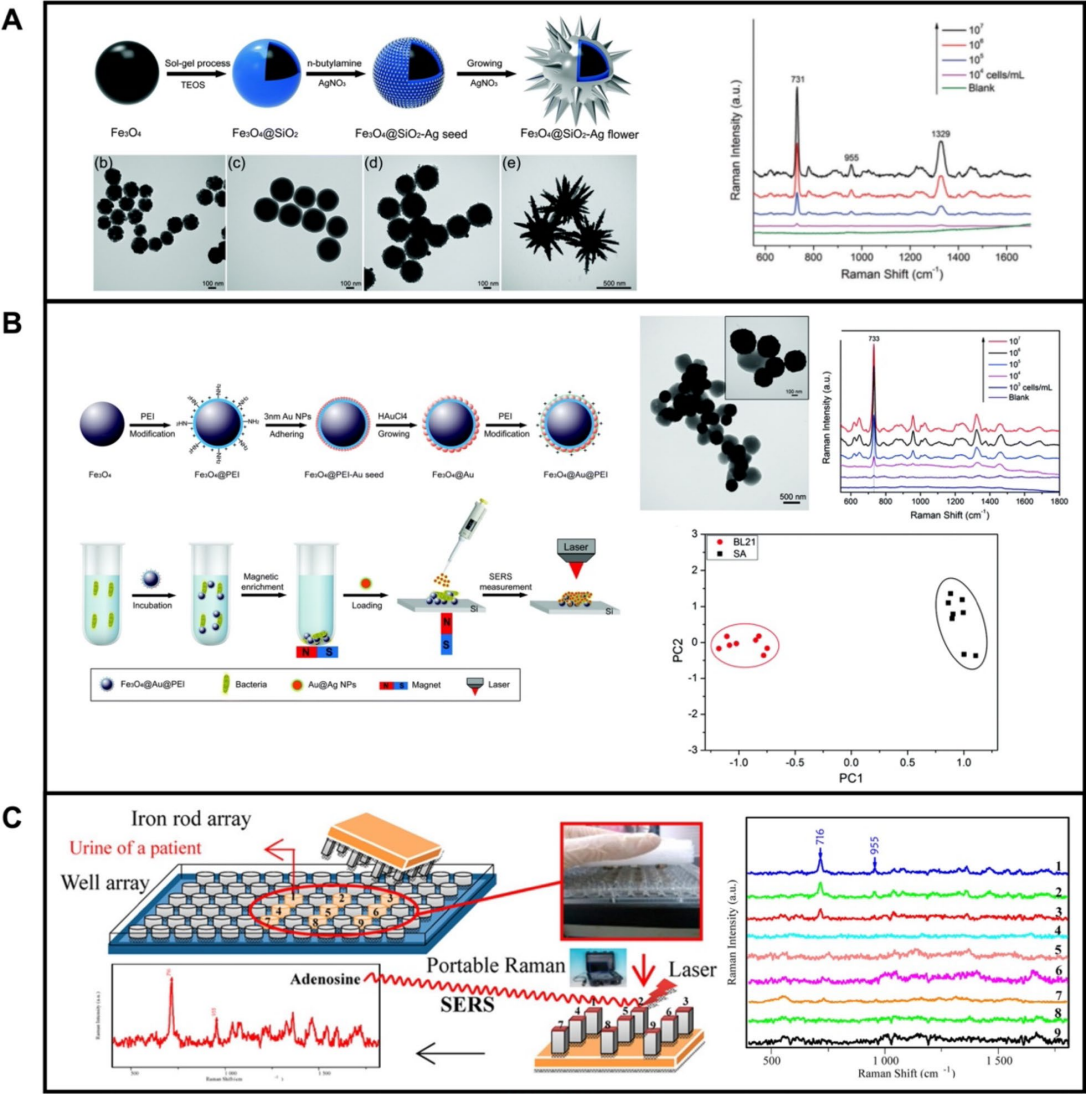
**A** Highly branched flower-like Fe_3_O_4_@SiO_2_@Ag nano-hybrid for bacteria detection: Schematic of the synthesis of Fe_3_O_4_@SiO_2_@Ag micro-flowers and corresponding SEM images at each step and SERS spectra for the detection of different concentrations of *Staphylococcus Aureus* using the nanohybrid; **B** Schematic of the synthesis of PEI-modified nanohybrids for the capture and enrichment of bacteria, SEM of Fe_3_O_4_@Au@PEI-*Escherichia coli* complex and corresponding SERS spectra and PCA differentiation between *Escherichia Coli* BL21 and *Staphylococcus Aureus* 24018. **C** Schematic illustration of Fe_3_O_4_/Au/Ag nano-hybrids for adenosine sensing and SERS spectra illustrating the detection with the sensing array (**B**) Reprinted with permission from ref. [171] (**A**); [95] (**B**) and [172] (**C**).

The use of magnetic actuated SERS substrates is beneficial for SERS sensing due to the facilities for simultaneous target capture and separation, interferences removal, and centrifugation avoiding thus interferences, especially from biological samples. The most widely used structures compromise a magnetic core and a metallic shell. Diverse types of magnetic materials have been used, including Fe_3_O_4_ [159, 160], MnFe_2_O_4_ [161], CoFe_2_O_4_ [162], Ni [163, 164], FePt [165] or CoPt [166]. The coating of the magnetic core with plasmonic nanoparticles can be performed by in-situ growth [167, 168] or ex-situ assembly [169, 170] methods. For example, Wang et al. [171] synthesized Fe_3_O_4_@SiO_2_@Ag nano-hybrid with a flower-like shape for capturing and sensing bacteria. As shown in Fig. 6A, SiO_2_ beads are coated with magnetic nanoparticles, followed by silver grown on their surfaces. Interestingly, by adjusting the amount of AgNO_3_, the structure of the nano-hybrid can be tailored into a micro-flower shape with a high degree of branches. Such design endows the nano-hybrid with superiorities: (a) excellent dispersion for improved response to the applied magnetic field; (b) larger surface area for enhanced target capture; (c) sharp tips from the branches for better hot spots in Raman signal enhancement; and (d) the possibility of sample preconcentration due to the presence of a magnetic Fe_3_O_4_ core. The resulting Fe_3_O_4_@SiO_2_@Ag nano-hybrid was modified with an aptamer for specific capture of *Staphylococcus Aureus* (Fig. 6A), with a limit of detection of 10^4^ cells per milliliter. In another example, Fe_3_O_4_@Au nano-hybrids were prepared by ex-situ assembly of 3 nm of AuNPs seed into Fe_3_O_4_ (Fig. 6B) [95]. By further coating the Fe_3_O_4_@Au with positive polyethyleneimine (PEI), the nano-hybrid can capture negative bacteria by electrostatic interactions, allowing for the capture and SERS-enhanced detection of *Escherichia Coli* and *Staphylococcus Aureus* bacteria. Related SEM measurements showed that Au surfaces of the nano-hybrid were attached to the bacterial wall, generating a Fe_3_O_4_@Au@PEI-bacteria complex. With the enrichment effect of the magnetic core and SERS activity outer Au surface, the LOD is 10^3^ cells per mL. The different Raman fingerprinting of the analytes can be subjected to principal component analysis (PCA) for the discrimination among bacteria. (Fig. 6B).

Yang et al. [172] used Fe_3_O_4_/Au/Ag nano-hybrids for adenosine sensing in a urine sample from lung cancer patients. To avoid interference from urea, an azo coupling reagent was employed to eliminate the urea (Fig. 6C). Such design allows for the fast determination of trace cancer biomarkers (adenosine) directly from urine samples (Fig. 6C).

### Non-plasmonic nanostructures as SERS substrates

**Fig. 7:**
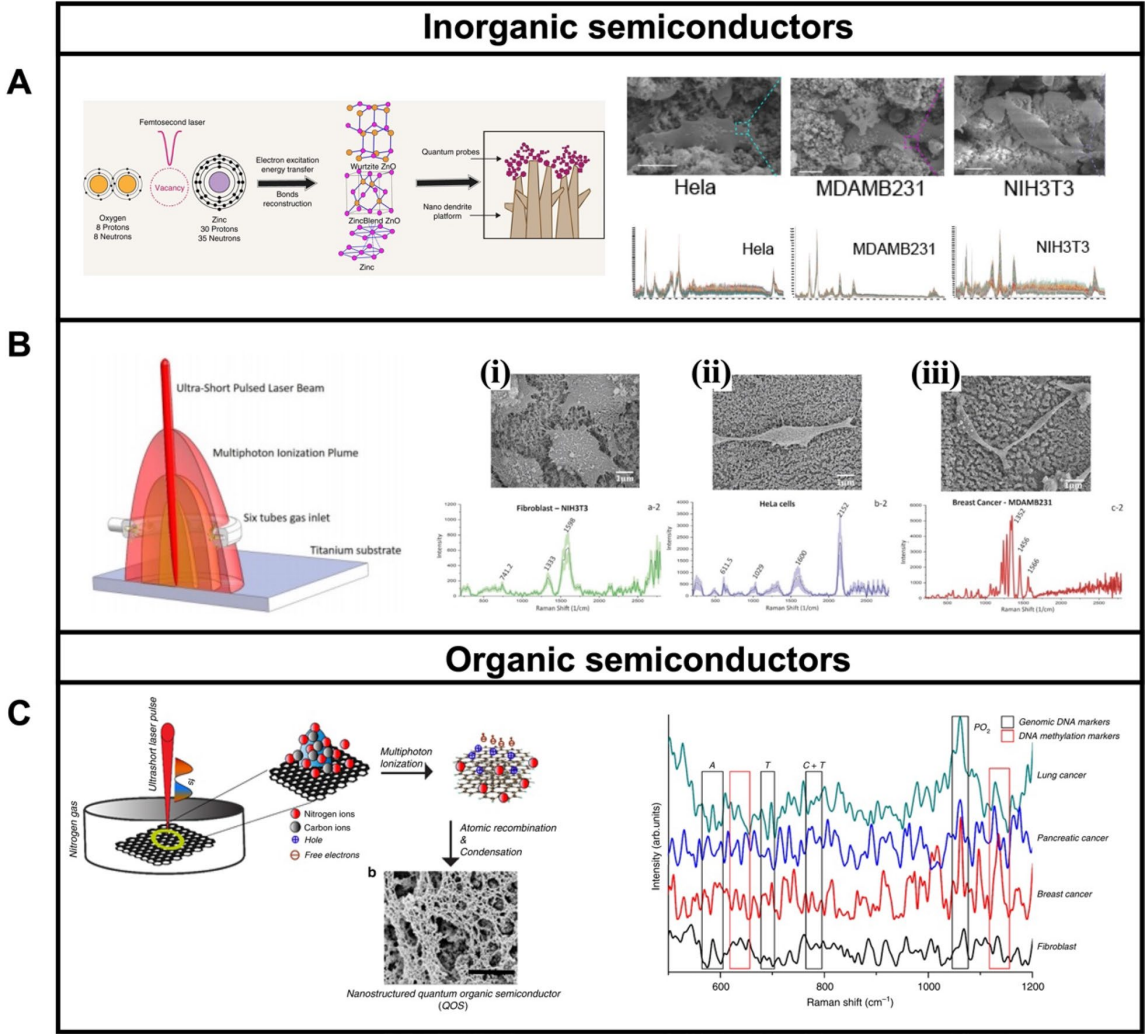
**A** Schematic of ZnO-based SERS substrates for in vitro cancer cell detection, SEM images illustrating the capture of Hela cells, breast cancer (MDAMB231) cells, and fibroblast (NIH3T3) cells and corresponding SERS spectra. **B** Inorganic semiconductor TiO_x_ as SERS substrates for cancer diagnostics: SEM images showing fibroblast-NIH3T3 cells (i), Hela cells (ii), and breast cancer-MDAMB231 cells (iii) capture TiO_x_ SERS substrate and corresponding SERS spectra. **C** Organic semiconductor SERS substrate for cancer stem cell makers sensing and SERS spectra of genomic DNA from diverse types of cells. Reprinted with permission from ref. [188] (**A**); [189] (**B**) and [192] (**C**)

Raman signal enhancement mechanisms based on EM enhancement employ plasmonic noble metals as SERS substrates [173, 174]. Semiconductor materials -either organic or inorganic- possess inherent Raman signals enhancement by charge-transfer mechanisms, paving a new way for SERS based on CM [175]. Advantages of the use of such nanomaterials include chemical stability, resistance to degradation, high absorptivity, and low cost [176–179]. Such properties endow the semiconductor-based SERS substrate with promising applications in cancer and pathogens cells or biomarker sensing. Inorganic semiconductors are based on solid-state structures of metal oxides [180, 181], metal sulfides [182, 183], metal halides [184, 185], and single elements [186, 187]. Haldavnekar et al. [188] fabricated ZnO-based semiconductor quantum probes for cancer cell’s SERS sensing. After performing femtosecond laser interaction, the size of the ZnO semiconductor was reduced to quantum scale (Fig. 7A), which is key to getting a high SERS activity with an enhancement factor of up to ~ 10^6^. The quantum scale semiconductors were combined with 3D nano-dendrite platforms for self-targeting, cell adhesion, and proliferation. The proposed platform was utilized for in-vitro sensing of two cancer cell lines. As depicted in the SEM images of Fig. 7A, Hela cells, breast cancer (MDAMB231) cells, and fibroblast (NIH3T3) can adhere to the nano-dendrite platform. The corresponding SERS spectra also showed distinct fingerprints for each type of cell. Keshavarz et al. [189] used multiphoton ionization growth on a Ti substrate, to fabricate TiO_x_ (Q-structured) nano particles restricted to the quantum scale (Fig. 7B). The resulting TiO_x_ semiconductors display high SERS activity with an enhancement factor of 3.4 × 10^7^. Figure 7B (right) shown the SEM morphology of fibroblast-NIH3T3 cell (i), Hela cell (ii), and breast cancer-MDAMB231 cell (iii) connected with the Q-structured TiO_x_ semiconductor and corresponding SERS spectra show the differences among the diverse types of cells.

The π-conjugated carbon structure of organic semiconductors imparts such materials with excellent biocompatibility to perform SERS sensing of biological samples [7, 190, 191]. Ganesh et al. [192] synthesized organic semiconductors as SERS substrates for investigating the epigenetic profile of cancer stem cells (Fig. 7C). The organic semiconductor is synthesized using an ultra-short, pulsed laser under a nitrogen gas environment, which enabled the shrinking of the organic semiconductor into a quantum scale. Such quantum scale endows the organic semiconductor with increased charge carrier mobility, which is necessary for efficient charge transfer in SERS. TEM observation revealed the particle size distribution of the organic semiconductor, with a diameter of 3.4 nm, which is in the quantum scale. Importantly, such organic semiconductors have high SERS activity with a 10^12^ enhancement factor. Epigenetic analysis of fibroblast cells (NIH3T3), breast cells (MDA-MB231), pancreatic cancer (AsPc-1), and lung cancer (H69-AR) cells was conducted. As shown in Fig. 7C (right part), genomic DNA of different cell lines had different peak intensities due to the differences in base composition.

### Indirect cancer and pathogens diagnosis based on SERS tags

The complexity of biological samples prevents direct SERS detection of a myriad of biomarkers, a cancer cell, or bacteria. To solve such a problem, indirect detection is performed using Raman tags that can interact specifically with the analyte, providing strong Raman signals. A representative SERS tag includes Raman enhancement nanomaterials, a Raman reporter, specific capture elements, and an internal standard [193, 194]. In some cases, SERS tags can experience random aggregation or degradation in complex samples at extreme pHs or ionic strength conditions. To avoid such a problem, the particle can be coated with silica sols [195] or polystyrene shells [196], for enhanced stability. Dopamine or SiO_2_ shells also can enhance the biocompatibility of SERS tags, thus reducing cytotoxicity in disease diagnosis in clinical samples [197, 198].

#### Raman reporter

Raman reporters are basic elements for designing efficient SERS tags and should possess the following characteristics: (A) stable and intense Raman signal; (B) clean spectral region with specific peaks; (C) ability to combine with Raman enhancement-related materials. Rhodamine (Rh6G) [199], crystal violet [196], malachite green [200], 5,5-dithiobis-2-nitrobenzoic acid (DTNB) [201], methylene blue (MB) [202], p-amino thiophenol (PATP) [203], etc., have been widely employed as Raman reporters for the fabrication of SERS tags.

#### Internal standard (IS)

An internal standard is a stable substance added in constant amounts to the samples and calibration solutions for reliable and quantitative detection. By the employment of an internal standard, the variations from the uneven EM or CM enhancement, as well as other inaccuracies such as instrumental variations will be reduced. IS used for SERS sensing should have the following properties: (a) uniform dispersion on SERS substrate [204]; (b) the signal intensities should be comparable to the sensing targets, with one band in the silent regions of the sensing target [205]. Different organic molecules such as β-mercaptoethylamine, 4-mercaptobenzoic acid, mercaptopyridine, etc., have been used as SERS internal standards [206]. Especially, Raman peaks from the SERS substrate can be used as IS. For example, Zou et al. [207] employed graphitic nanomaterials as IS due to their unique, strong, and stable Raman peaks localized in the silent Raman region of the sensing targets. Zhang et al. [208] fabricated carbon nanotube/Ag nanoparticle composites (CNT/AgNPs) as SERS substrates, with carbon nanotube acting as IS for the calibration of the intensity of the SERS targets. As a result, the integration of IS into the SERS substrate provides a reliable way for quantitative SERS detection of targets, which is important for the realization of clinical applications.

#### SERS Tags

By incorporating a Raman reporter, the SERS tag can provide a Raman signal with high stability and intensity. Specific capture probes can be used for target delivery and interaction with the analyte of interest. The capture element can be categorized into the following groups [1, 27, 209]: (A) antibody, which is a Y-shaped protein that is widely used in recognizing antigens with high efficiency and specificity. However, they always suffer from low stability and high cost [210, 211]; (B) aptamer, which is DNA or RNA that can specifically bind to the target, acting as a cheap, stable, and highly specific recognition element. Yet, the limited selection for targets, cross-reactivity and stability issues (due to its small size) makes aptamers much less popular than antibodies, especially for clinical applications [212, 213]; (C) molecularly imprinted polymer (MIPs), which are prepared by using molecular imprinting technique, leaving cavities in the polymer with high specificity for the chosen “template” molecule. Such recognition elements are cheap and can be used in compartments at extreme (pH, temperature) conditions [214]; (D) antibiotic, which is used for the inactivating/killing of bacteria. Such capture elements also possess superiorities such as cheap, chemically stable, and high production rate, still, they are only special for the recognition of different types of bacteria, with limited selectivity for different bacterial strains [215]; (E) other recognition elements [216–218] such as transferrin are also used for the targets capture due to the overexpression of related receptors on cancer cell surfaces. Cancer and pathogens diagnosis strategies using several types of recognition elements and SERS detection are listed in Table 4.

**Table 4:** SERS tags using different specific recognition elements for indirect cancer and pathogens diagnosis

Recognition elements	SERS Tags		Reporter	Target analyte/cells	Refs.
	Structure	Morphology			
Antibody	Antibody-DTNB-Au nanorod		DTNB	*Staphylococcus aureus*	[210]
	Antibody-DTNB-Au@Ag nanorod		DTNB	Circulating tumor cells	[211]
Aptamer	Aptamer- conjugated Au		DTNB, MBA	*Escherichia Coli* *Staphylococcus* *aureus*	[212]
	Aptamer-DSPE-graphene isolated Au nanocrystals (GLANs)		2D carbon nanomaterials	HepG2, A549 cell lines	[213]
MIPs	MIP-PATP-Ag nanoparticles		PATP	Sialic acid (cancer cells and tissues imaging)	[214]
Antibiotic	Vancomycin (Van)-MBA-Au		MBA	*Staphylococcus aureus*	[215]
Luteinizing hormone-releasing hormone (LHRH)	LHRH-pMBA-Au nanoparticles		4-mercaptobenzoic acid (pMBA)	Circulating tumor cells	[216]
Transferrin	Transferrin-BDT-Au		1,4-benzenedi-thiol (BDT)	Hela cell	[217]
Folic acid	FA-Au@polyPOPA@Ag		Rh6G MB	Human lung adenocarcinoma cell line A549	[218]

**Fig. 8:**
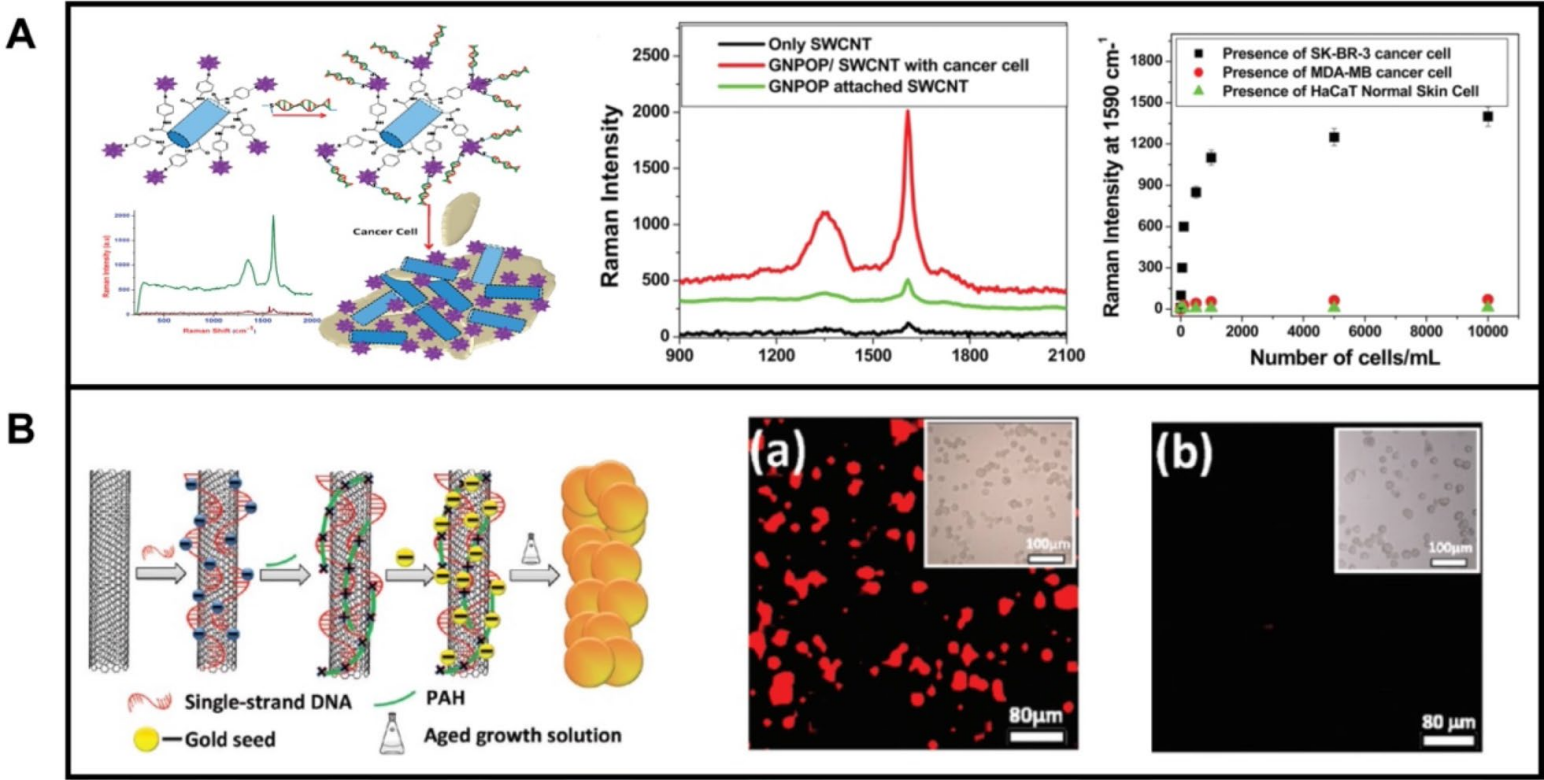
**A** Au nano popcorn modified SWNCTs nano-hybrid for specific diagnosis of SK-BR-3 cancer cells. The left part shows the schematic illustration in the synthesis procedure its application in cancer cell diagnosis; middle part shows the Raman spectra of SWCNTs, Au nano popcorn modified SWCNTs nano-hybrid, and Au nano popcorn modified SWCNTs nanocomposites decorated with SK-BR-3 cancer cell and the right part the SERS intensity changes at 1590 cm^−1^ after the addition of different amounts of SK-BR-3, MDA-MB human breast cancerous, and HaCaT normal skin cells to the aptamer modified nano-hybrid. **B** Schematic illustration of the synthesis of SWNCT-Ag-PEG-FA or SWNCT-Au-PEG-FA nano-hybrids for specific Raman imaging of KB cells and Raman images of SWNCT-Au-PEG-FA incubated with KB cells (middle part) and Hela cell (right part). Reprinted with permission from ref. [219] (**A**) and [220] (**B**)

Beqa et al. [219] prepared Au nano-popcorn/SWCNTs nanohybrids for theragnostic applications. Figure 8A shows the schematic of the fabrication process, in which SWCNTs were functionalized with sulfydryl- groups, followed by Au nano popcorn attachment onto the nanotube surfaces via an Au–S bond. In the following step, the S6 aptamer with sulfhydryl- group was attached to the nano-hybrid. This nano-hybrid show superior performance in the sensing of SK-BR-3 cancer cells. After Au nano-popcorn were adsorbed onto the SWCNTs surfaces, the Raman signal from SWCNTs (D band, 1300 cm^−1^; G band, 1590 cm^−1^) increased by an order of magnitude. Importantly, the SK-BR-3 cancer cell can induce the aggregation of the aptamer-modified nano-hybrid to generate hot spots. The strategy is highly specific as revealed in the low Raman signal enhancements in the presence of other cells (MDA-MB or HaCaT normal skin cells). Wang et al. [220] modified SWNCTs with Ag or AuNPs for cancer cell imaging. As depicted in Fig. 8B, the SWCNTs were modified with DNA, followed by seed growth of the NPs to generate the SWCNT-Ag or SWCNT-Au nano-hybrids. The surfaces of the as-prepared nano-hybrids were further modified with polyethylene glycol (PEG) to improve the stability in physiological conditions. Next, the authors modified the nano-hybrid with folic acid (FA) for specific attachment of human carcinoma KB cells and cell imaging. As expected, KB cells incubated with FA-modified nano-hybrid showed apparent Raman signals, while Hela cells show no obvious Raman signals.

## Clinical translation of SERS-based cancer and pathogens diagnosis: current advantages and challenges

### Enhanced robustness and reproducibility of Raman signal enhancement

One important limitation of SERS detection that hampers the translation from laboratory to the clinical setting is their robustness and reproducibility. Significant efforts had been aimed to address this issue through the following methods: (1) The introduction of ordered SERS substrates. A SERS substrate with a highly regular structure is the key to producing uniform Raman signal enhancement, ensuring thus the accuracy and reproducibility in clinical applications. For instance, Kim et al. [221] sputtered a monolayer polystyrene nanosphere with a gold layer to form regularly arranged nanostructures. The periodic structure can function as a SERS substrate with < 5% relative standard deviation for high reliability and reproducibility. Later, tear fluids from breast cancer patients and control groups were measured by a handheld Raman spectrometer with the as-fabricated periodic SERS substrate, with a clinical sensitivity of 92% and a specificity of 100% for breast cancer identification. Yet, it is unclear if the biomarkers are related to cancer, since further validation is missing. Zhu et al. [222] employed periodic arrays (hexagonal-packed gold film over nanosphere, AuFON) to improve the reproducibility of detection. In addition, the hydrophilic/hydrophobic character of the SERS substrate endows the platform with reduced nonspecific adsorption, which is important for lateral accurate and sensitive detection. Direct detection in blood can be achieved. (2) The introduction of SERS tags. The encoding of Raman reporters into the plasmonic metal to function as SERS tags could improve the stability, specificity, and uniformity of Raman signal enhancement. Bai et al. [223] designed SERS tags using AuNPs as SERS substrate and 3 different molecules with specific narrow Raman bands in the bio-silent region as reporters. With further antibody modification, the immunoassay of 3 different liver cancer antigens including α-fetoprotein (AFP), carcinoembryonic antigen (CEA), and ferritin (FER) was conducted in 39 clinical serum samples. (3) The introduction of IS. A reliable quantitative detection method also is necessary for clinical applications. To address this issue, the employment of an internal standard could effectively calibrate the variations from the uneven EM or CM enhancement, as well as other inaccuracies such as instrumental variations. Lin et al. [224] developed a label-free SERS-based method for DNA detection. SERS signals from the phosphate backbone were used as IS to improve the accuracy in the quantitative analysis of DNA. Such a method has been successfully employed for circulating DNA sensing, with good sensitivity and specificity for differentiating nasopharyngeal cancer patients from normal subjects.

### Portable Raman spectrometers for clinical translation

**Fig. 9:**
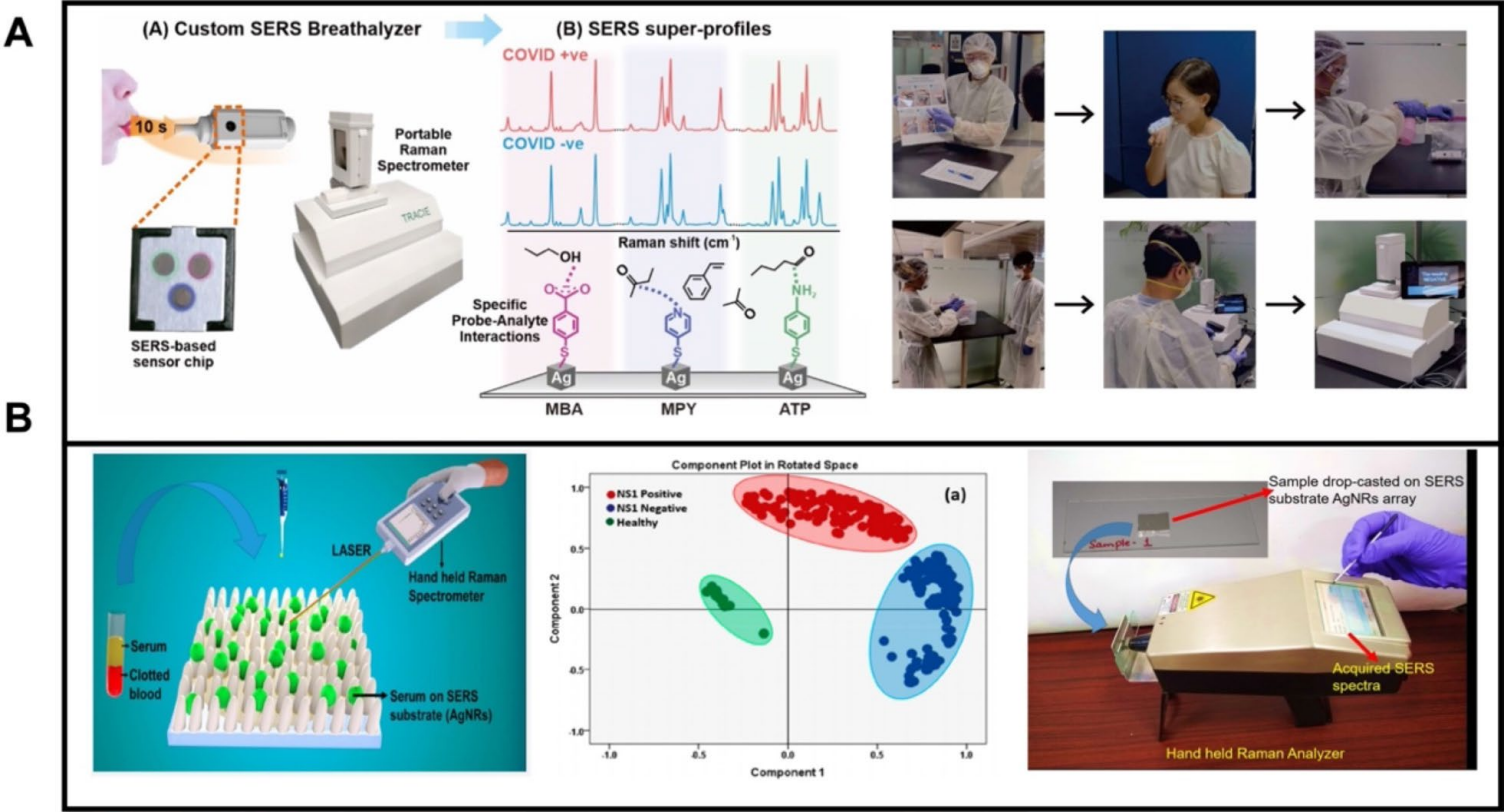
**A** SERS-based breathalyzer for mass screening of COVID-19, schematic of the interaction of different molecular receptors interacts with BVOCs, and photographs describing the participant recruitment workflow. **B** Silver nanorod array for dengue diagnosis in blood samples using a hand-held Raman spectrometer and PCA discrimination. Reprinted with permission from ref. [225] (**A**) and [32] (**B**)

Traditional Raman measurements for SERS detection needs to be conducted in a special laboratory due to the enormous size of the equipment, which will restrict its real applications for clinical translation. As a solution for portable detection, Leong et al. [225] developed a hand-held SERS breathalyzer to identify Coronavirus disease 2019 (COVID-19) individuals through the detection of volatile organic compounds (BVOCs) in breath (Fig. 9A). Multiple molecular receptors were employed to capture BVOCs in exhaled breath and acquire specific spectral variations between COVID-positive and COVID-negative individuals (middle part). The SERS-based breathalyzer allows for fast and noninvasive screening with high sensitivity and specificity for COVID-19 detection. Gahlaut et al. [32], developed a SERS-based diagnosis of dengue virus in blood samples. Silver nanorod array were used as SERS substrates (Fig. 9B). With the employment of a hand-held Raman spectrometer, such detection could be conducted in the field wherever needed, which is important in the clinical diagnosis (right part). They also demonstrate SERS diagnosis of dengue virus in blood samples collected from 102 subjects, successfully validating the approach.

### Machine learning methods for clinical translation

**Fig. 10:**
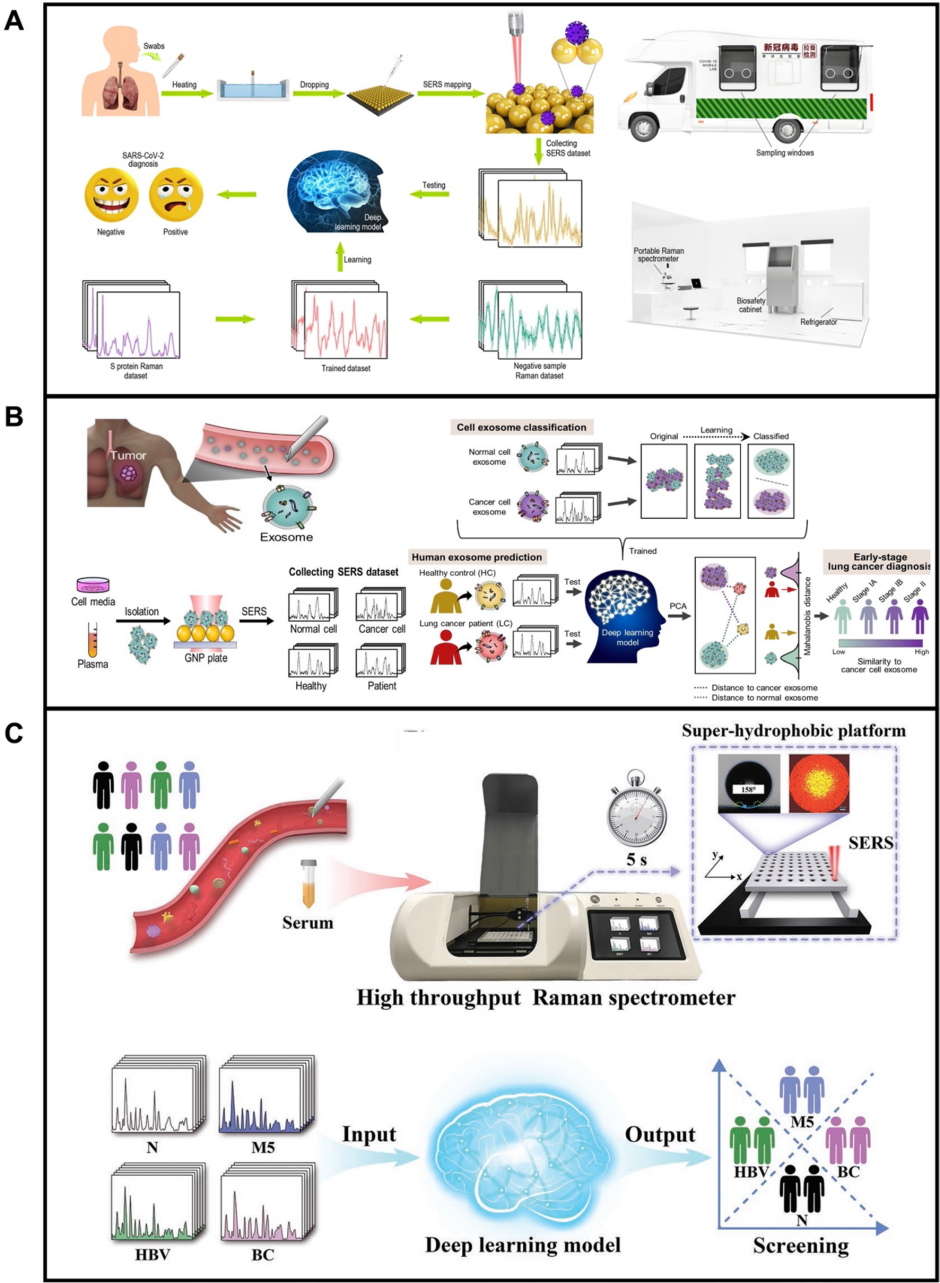
**A** Detection of SARS-CoV-2 antigens by deep learning-based SERS technology. The left part is a schematic illustration of the working flow; the right part is a schematic illustration of the SERS mobile detection platform. **B** Deep learning-based circulating exosome analysis for lung cancer diagnosis, left part show circulation of lung cancer tumor exosomes in the bloodstream and schematic illustration of the collection of SERS spectroscopy of exosomes; right part reveals that spectral dataset is used to train the deep learning models. Reprinted with permission from ref. [229] (**A**) and[230](**B**) and [231] (**C**)

In the direct detection of cancer or pathogens, specific fingerprints from the vibration of molecules may suffer from subtle differences which are difficult to be identified by manual visual inspection. Therefore, machine learning which is part of artificial intelligence (AI), has been integrated into SERS detection to identify features or perform classification. Due to the power of machine learning methods, information could be extracted effectively from the vibrational spectra of complex mixtures or big datasets, which promotes the translation of SERS from proof-of-concept to clinical applications [226]. The most usual form of machine learning is the supervised learning methods, which include a discriminant analysis-based method, artificial neural network, k-nearest neighbor, etc. This model learns by extracting knowledge from the raw data and using this as-obtained knowledge to make decisions from unknown samples [227]. Unsupervised learning algorithms have been also introduced and compared with their supervised counterparts [228]. Huang et al. [229] employed AuNPs array as SERS substrates for label-free detection of SARS-Cov-2. Due to the nail-like shape of the virus, the spike (S) protein has the maximum probability to interact with the hot SERS spots and was thus chosen as the detection target. In the lateral clinical applications, a deep-learning algorithm of pure S protein and negative clinical specimens was used in the rapid screening of SARS-CoV-2 antigen, with an identification accuracy of 87.7% (Fig. 10A). In addition, a portable Raman spectrometer was used to conduct the detection, pre-treatment, and spectra measurements. While promising, the overall accuracy of the detection should be improved. Shin et al. [230] used a deep learning-based computer algorithm to overcome the complexity and heterogeneity in analyzing the SERS signal of exosomes in blood (Fig. 10B). After collecting the Raman signals of exosomes using an AuNPs-coated plate as a SERS substrate, the as-obtained spectral dataset is used to train the deep learning models (right part). In a prediction of 43 cancer patients, they also show good results by the integration of SERS analysis and deep learning model, which means a promising method for the early-stage liquid biopsy of lung cancer. Still, the method is limited by the lack of validation studies or additional biomarker identification. Because circulating biomarkers from tumors in these biofluids are very scarce and most biomolecules are from non-tumor origins, careful considerations should be made to ensure the separation is not due to overfitting*.* Lin et al. [231] employed super-hydrophobic substrates in connection with deep learning techniques for developing high throughput, a label-free analytical platform for disease screening (Fig. 10C). In this work, an aluminum plate with a super-hydrophobic groove was specially designed to prevent the coffee ring effect and promote the self-localization of the droplet. The SERS platform was then applied for serum sample detection containing breast cancer (BC), hepatitis B virus (HBV), and leukemia M5 (M5) patients. The deep learning model was trained to statistical analysis the SERS spectral data from clinical samples to classify large and complicated fingerprints of target molecules, which is important for efficient large-scale population cancer screening.

During the past years we have witnessed the significant effort to translate SERS approaches from the laboratory to clinical use, which is a promising option for future cancer and pathogen detection. Yet, critical issues still need to be considered: (1) Direct measurements have the advantage of showing some chemical information, yet related works fail to provide what biomarkers were detected, which are important to explain why these changes in Raman peaks are related to diseases, as well as the further validation of the findings. Therefore, further studies related to direct detection should go deep into the identification of related biomarkers and include interdisciplinary validation studies. (2) In promoting clinical translation, even though SERS substrates are designed into periodic structures to improve the uniformity for Raman signal enhancement, massive cancer, or pathogen disease screening is restricted to the small cohort sizes of the SERS substrate. Hence, scientists should focus more on the fabrication of large-scale periodic SERS substrates. (3) Recent progress proved that SERS could be utilized in large-scale COVID-19 screening, with fast, high sensitivity and specificity. Yet, sample collection by breathalyzer may face drawbacks. Compare with traditional throat swabs where sample collection is conducted by the doctor, participants will need to conduct the breathalyzer themself where the doctors must explain what they need to do. It is also hard to ensure the amounts of exhales to keep similar among different people. In addition, so many people blowing at the same place may increase the risk of cross-infectious.

## Conclusion and prospect

Cancer and pathogens are now the major causes of death around the world, thus the development of simple, rapid, and effective diagnostic tools. Driven by the development of optical and material science, SERS have shown immense potential for disease diagnosis, including cancer cells, microorganism, and related biomarkers sensing. In this review, we have discussed recent advances in SERS-based strategies for cancer and pathogens diagnosis via direct and indirect ways. In the direct detection mode, despite SERS-based strategies have provided quick, sensitive, and basic chemical structures of target molecules for disease diagnosis, Raman signal enhancement is an overly complex phenomenon and depend on the nanomaterials size, aggregation degree, and analyte/substrate interaction mode. Therefore, the clinical application needs to focus on the reproducibility of signal output in the quantification of disease targets. As signal amplification of analyte depends on the strength of the EM field that is distributed around plasmonic nanomaterials with remarkable differences, even the same molecule close to different positions of the nanomaterials will lead to different signal intensity output. In addition, EM field distribution from different signal nanoparticles interacts with each other when two nanoparticles are getting close to a specific distance. Hence, the EM field distribution of whole aggravated nanoparticles is different from that arising from a single nanoparticle [232–234]. The use of IS is another strategy to simplify quantitative SERS analysis, in which unstable signals from the inhomogeneous distribution of the EM field will be corrected by the internal peaks [207, 235, 236]. Direct SERS detection also provides chemical information of the targets, which could function as a “*finger-print*” for direct bacteria or cancer cell identification. Yet in cases, the subtle differences from their specific Raman spectra are hard to identify. To solve this problem, machine learning has been used to identify features or perform classification, in which information could be extracted effectively from the complex mixtures or big datasets, thus helping in disease diagnosis or screening in clinical applications [229, 230, 237, 238]. In the indirect detection mode, Raman reporters, enhancement nanomaterials, vibrational spectra, and specific recognition elements are integrated into one SERS tag, which can capture targets and provide strong and identical Raman signals with high sensitivity and specificity. Though the indirect detection mode will lose the natural information of chemicals, the employment of SERS tags enables the promising detection of targets from complex real samples, which is important for clinical applications [223, 239].

Another important challenge that still requires attention is the biocompatibility of SERS substrates for living cancer and pathogens cells imaging, or in-vitro analyte monitoring. The most used plasmonic metal (Ag, Au, Cu, etc.) possess high cytotoxicity and thus may cause changes in cell structures (target protein or other organic chemicals) and influence the mapping/sensing results. The coating of metal nanomaterials with biocompatible materials such as polyethylene glycols [240], SiO_2_ [198], etc. can reduce their cytotoxicity. However, the coating of the biocompatible membrane may hamper the Raman enhancement ability of the SERS substrate [241]. Integration of carbon materials such as graphene also can reduce the cytotoxicity of metals [242], still, the total size of nano-hybrid needs to be considered due to the optimal nanomaterial radius for endocytosis is about 25–30 nm [243]. Therefore, further efforts still should have focused on the fabrication of SERS substrates with good biocompatibility and SERS activity.

The development of portable and miniature SERS platforms is also important to expand the applicability of disease diagnosis with higher realistic scenarios. Current attempts such as portable Raman devices [244] and microfluidic platforms [245], etc., make disease diagnosis more flexible, still, traditional SERS techniques are widely used in cancer and pathogens diagnosis due to their superior SERS performance such as sensitivity, spectral resolution, and mapping functionality. As detail described above, portable devices already have been successfully utilized in the screening of COVID-19 individuals in clinical breath samples or dengue virus in clinical blood samples. Such works have fully proven the effectiveness of portable devices in the realization of clinical translation, as well as enormous potential in the development of noninvasive human diagnostic tools for point-of-care detection or mass screening purposes. Therefore, further efforts still need to focus on realizing disease diagnosis in a portable way to meet clinical needs.

Lastly, the combination of SERS and other technologies also shows great potential in promoting SERS detection from proof-of-concept to clinical translation, which includes: (1) The coupling between electrochemistry (EC) and SERS, in which chemical enhancement could be strengthened by EC, as well as molecules may be captured (while molecular charge and electrode potential are at appropriate state) by substrate/electrode to get closer to the strong electromagnetic field, hence results in higher Raman signal enhancement. Importantly, the integration of EC makes molecular adsorption on substrate/electrode with high uniformity and brief time, which is important for further quantitative analysis and clinical applications [246]. (2) The integration of SERS onto a flexible substrate to function as wearable sensors. Wearable sensors have made great progress in recent years. Due to their advantages such as the capability of remote monitoring and continuous detection, as well as high biocompatibility and wearability, wearable devices also show great promise in advancing SERS into clinical applications [247, 248]. (3) The employment of micro/nanomotor for enhanced sensitive and rapid SERS detection. Current SERS substrates mostly can only interact with targets through free diffusion of the molecules or nanoparticles themselves which would limit the effective recognition between the analyte and related active surfaces, thus resulting in confined detection speed and sensitivity. The micro/nanomotor are fabricated micro/nano actuators that could transform outside energies into their mechanical motion. Diverse types of energy such as chemical energy, light, ultrasound, or magnetic energy could be used in micro/nanomotor propulsion. By the integration of micro/nanomotor, the above-mentioned problems in SERS detection would be well solved and help in the realization of clinical translation [249, 250].

## Data Availability

Not applicable.

## Abbreviations

SERS: Surface enhanced Raman scattering
EM: Electromagnetic enhancement
CM: Chemical enhancement
EF: Enhancement factor
MoS_2_: Molybdenum disulfide
3D: Three dimension
SEM: Scanning-electron microscopy
LOD: Limit of detection
EV71: Enterovirus 71
TEM: Transmission electron microscopes
DENV: Dengue virus
AuNRs: Gold nanorods
PEG: Polyethylene glycol
RGD: Arg-Gly-Asp
NLS: Nuclear localization signal
NIR: Near infrared spectroscopy
CFU: Colony-forming unit
PEI: Polyetherimide
RNA: Ribonucleic acid
DNA: Deoxyribonucleic acid
NPs: Nanoparticles
*E.coli*: *Escherichia coli*
BN: Boron nitride
BP: Black phosphorous
G@AgNPs@Si: Graphene-silver nanoparticles-silicon
Ag@NGO: Graphene oxide coated with silver nanoparticles
WS_2_: Tungsten disulfide
g-C_3_N_4_: Graphitic carbon nitride
CNTs: Carbon nanotubes
SWCNTs: Single-wall carbon nanotubes
MWCNTs: Multi-wall carbon nanotubes
FA: Folic acid
C-dot-Ag-NP: Carbon-dot/silver-nanoparticle
PDMS: Polydimethylsiloxane
C-dots: Carbon dots
HAuCl_4_: Chloroauric acid
ATP: Adenosine triphosphate
NSL: Nanosphere lithography
EBL: Electron beam lithography
CTCs: Circulating tumor cells
RSV: Respiratory viruses
PCV2: Porcine circovirus 2
PRV: Pseudorabies virus
H5N1: Influenza A
Fe_3_O_4_: Iron Oxide
CuO: Copper(II) oxide
MnFe_2_O_4_: Manganese Iron Oxide
ZnO: Zinc oxide
CoFe_2_O_4_: Ni: Nickel
FePt: Iron-platinum
CoPt: Cobalt–platinum
SiO_2_: Silicon dioxide
PCA: Principal component analysis
Rh6G: Rhodamine 6G
DTNB: 5,5-Dithiobis-2-nitrobenzoic acid
MB: Methylene blue
PATP: P-aminothiophenol
MIPs: Molecularly imprinted polymer
pMBA: 4-Mercaptobenzoic acid
BDT: 1,4-Benzenedi-thiol
LHRH: Luteinizing hormone releasing hormone
GLANs: Aptamer-DSPE-graphene isolated Au nanocrystals
